# Wood Growth in Pure and Mixed *Quercus ilex* L. Forests: Drought Influence Depends on Site Conditions

**DOI:** 10.3389/fpls.2019.00397

**Published:** 2019-04-02

**Authors:** Enrica Zalloni, Giovanna Battipaglia, Paolo Cherubini, Matthias Saurer, Veronica De Micco

**Affiliations:** ^1^Department of Agricultural Sciences, University of Naples Federico II, Naples, Italy; ^2^Department of Environmental, Biological and Pharmaceutical Sciences and Technologies, University of Campania “Luigi Vanvitelli”, Caserta, Italy; ^3^Swiss Federal Research Institute WSL, Zurich, Switzerland; ^4^Department of Forest and Conservation, Sciences Forest Sciences Center, The University of British Columbia, Vancouver, BC, Canada

**Keywords:** mediterranean, tree rings, water use efficiency, δ^13^C, δ^18^O, basal area increment

## Abstract

Climate response of tree-species growth may be influenced by intra- and inter-specific interactions. The different physiological strategies of stress response and resource use among species may lead to different levels of competition and/or complementarity, likely changing in space and time according to climatic conditions. Investigating the drivers of inter- and intra-specific interactions under a changing climate is important when managing mixed and pure stands, especially in a climate change hot spot such as the Mediterranean basin. Mediterranean tree rings show intra-annual density fluctuations (IADFs): the links among their occurrence, anatomical traits, wood growth and stable isotope ratios can help understanding tree physiological responses to drought. In this study, we compared wood production and tree-ring traits in *Quercus ilex* L. dominant trees growing in two pure and two mixed stands with *Pinus pinea* at two sites in Southern Italy, on the basis of the temporal variation of cumulative basal area, intrinsic water use efficiency (WUE_i_), δ^18^O and IADF frequency in long tree-ring chronologies. The general aim was to assess whether *Q. ilex* trees growing in pure or mixed stands have a different wood production through time, depending on climatic conditions and stand structure. The occurrence of dry climatic conditions triggered opposite complementarity interactions for *Q. ilex* growing with *P. pinea* trees at the two sites. Competitive reduction was experienced at the T site characterized by higher soil water holding capacity (WHC), lower stand density and less steep slope than the S site; on the opposite, high competition occurred at S site. The observed difference in wood growth was accompanied by a higher WUE_i_ due to a higher photosynthetic rate at the T site, while by a tighter stomatal control in mixed stand of S site. IADF frequency in *Q. ilex* tree rings was linked to higher WUEi, thus to stressful conditions and could be interpreted as strategy to cope with dry periods, independently from the different wood growth. Considering the forecasted water shortage, inter-specific competition should be reduced in denser stands of *Q. ilex* mixed with *P. pinea*. Such findings have important implications for forest management of mixed and pure *Q. ilex* forests.

## Introduction

Wood growth in mixed vs. pure stands depends on several factors, such as species composition, stand density, age and climatic conditions (Forrester, 2014). During the development of mixed stands, there might be changes in the dominance of species with different growth and physiological strategies (Forrester, 2015). Interactions between species could be ascribed to competition, with a negative effect of one species on the other, to competitive reduction, when inter-specific competition is less than intra-specific one because of a differentiation in resource use strategies, or to facilitation, with a positive effect of one species on the other (Forrester, 2014). Many studies have shown that mixed stands under stressful conditions (e.g., very high stand density, poor water supply, drought or nutrient shortage), are more productive than pure ones (Amoroso and Turnblom, 2006; Erickson et al., 2009; Pretzsch et al., 2013a,b; del Río et al., 2014), following the assumption of the stress gradient hypothesis (SGH). SGH hypothesis suggests that facilitation is favored when the environmental conditions become harsher (Bertness and Callaway, 1994). However, this is not always the case and mixed stands are not always better adapted to climate constraints if compared to monospecific stands. Complementarity, which is the set of competition and facilitation interactions possibly occurring in mixed and pure populations, may show variations due to climatic factors depending on: the different species reactivity to stand density (Forrester et al., 2013), tree size (Forrester, 2015), site conditions (Binkley, 2003; Pretzsch et al., 2010; Coates et al., 2013; Dieler and Pretzsch, 2013), microclimatic differences (Lebourgeois et al., 2013), and the severity of climatic extremes.

The Mediterranean region is foreseen to be strongly affected by global warming, leading to enhanced drought stress for trees in many ecosystems (Giorgi, 2006; Somot et al., 2008; IPCC, 2017). The increased intra-annual frequency and duration of drought periods in the Mediterranean Basin may lead to changes in water use efficiency (WUE) depending on the species, stand density, tree size and age, and growth rate (Brienen et al., 2017). The latter is reported to scale positively with WUE (Huxman et al., 2008). However, fast-growing trees in mixed stands could suffer from drought more than slower growing trees in monocoltures since they generally use more water (Law et al., 2002; Schume et al., 2004; Forrester, 2015). Tree responses to the changing environmental conditions can be reconstructed with the study of tree-ring features in chronologies of tree-ring width, anatomical traits or stable isotope composition (McCarroll and Loader, 2004; Čufar, 2007; Fonti et al., 2010). The combination of carbon and oxygen stable isotope analysis with tree-ring growth provides information about tree ecophysiological processes in response to stress, suggesting which physiological process, namely carbon uptake or water loss, prevailed in determining the variation in WUEi (Scheidegger et al., 2000), expecially in severely water-limited ecosystems (Gessler et al., 2014; Altieri et al., 2015; Moreno-Gutiérrez et al., 2015; Battipaglia et al., 2016b). Grossiord et al. (2014a,b) found that the stand-level δ^13^C declined with increasing diversity in temperate beech and thermophilous deciduous forests but not in hemiboreal, mountainous beech and Mediterranean forests. Within the Mediterranean region, studies analyzing the complementarity effects between mixed and pure stands are scarce. Grossiord et al. (2014c) found that *Quercus cerris* L. trees did not reduce transpiration in response to drought when growing in pure stands, but significantly reduced transpiration and increased WUE_i_ in mixed stands with *Quercus petraea* (Mattuschka) Liebl. Battipaglia et al. (2017) showed a higher wood productivity and WUE in mixed stands of *Quercus robur* L. and *Alnus cordata* Loisel. in comparison with *Q. robur* pure stands, due to the positive N-fixation effect of *A. cordata*. Understanding which factors drive inter- and intra-specific interactions under a changing climate is necessary when managing mixed and pure stands, since one of the priority in forestry is to acquire knowledge on the capability of different forest ecosystems to adapt to short- and long-term climatic variability (Brooker, 2006), especially in so called climate-change hot spots such as the Mediterranean. *Quercus ilex* L. forests widely occur throughout the Mediterranean basin, both in pure stands or in mixed forests with Mediterranean pines such as *Pinus pinea* L. (Terradas, 1999), differing in light demand, root system and physiological strategies in response to drought. It is still unknown whether mixed stands would be more capable to acclimate to forecasted increase in intra-annual climate variability in the Mediterranean, if compared to pure stands.

Mediterranean trees often form peculiar anatomical traits in tree rings called intra-annual density fluctuations (IADFs), which have been linked to intra-annual frequency of dry periods (De Micco et al., 2016). They have been considered either an hydraulic adjustment of trees to drought or a strategy to take advantage of favorable conditions of growth after a drought event (Battipaglia et al., 2016a). Finding the link between IADF occurrence and facilitation or competition effects at different sites, under different micro-climatic conditions, may be useful to add insights on the ecological role of these tree-ring traits. In this study, we aimed to (1) analyze the dynamics of complementarity effects of *Q. ilex* dominant trees growing in a pure and in a mixed stand with *P. pinea* at two study sites differing for tree age, stand density, slope and soil characteristics, on the basis of tree-ring growth and stable isotope ratio variations, (2) analyze the different tree growth response to climatic factors, (3) find the link between wood anatomical recurrent traits, such as IADFs and tree growth in the different study sites. In order to reach these aims, we investigated the temporal variation of cumulative basal area, intrinsic WUE (WUE_i_) assessed through δ^13^C and δ^18^O in tree rings (Moreno-Gutiérrez et al., 2012; Altieri et al., 2015; Battipaglia et al., 2016b), in each pure stand in comparison with mixed ones, calculating annual indexes of complementarity. We hypothesize that: (1) *Q. ilex* tree growth is higher in pure than in *P. pinea*-mixed stands, accompanied by a higher WUE_i_; (2) precipitation is the main factor influencing *Q. ilex* tree growth at all the Mediterranean study sites; (3) IADFs occur where wood growth is lower, because linked to stressful conditions of growth rather than to favorable ones.

## Materials and Methods

### Study Sites

**FIGURE 1:**
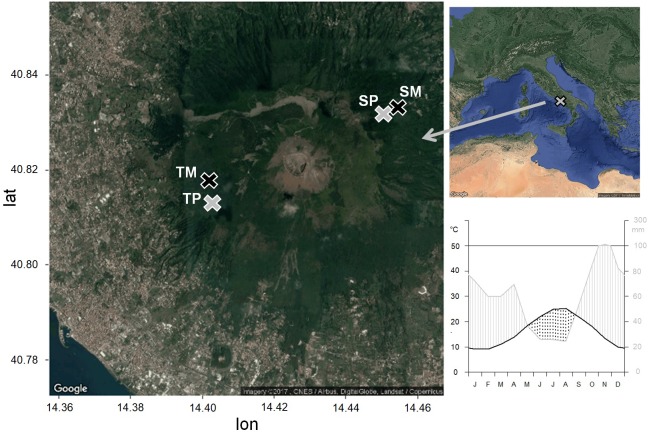
Location of the study sites and climatic diagram of the period 1985–2005, from the CRU TS3.23 gridded dataset at 0.5° resolution data (Kahle and Wickham, 2013). Pure stands are in gray, mixed stands are in black. TP, tirone alto vesuvio pure stand; TM, tirone alto vesuvio mixed stand; SP, somma pure stand; SM, somma mixed stand.

The study sites are located within the Mediterranean region, in the Vesuvio National Park, southeast from Naples, Southern Italy. The two sites are located on two opposite slopes, one in the southwest-faced “Tirone Alto-Vesuvio” Forest State Reserve and the other on the northeast-faced Mount Somma slopes, differing by stand density, slope, aspect (Table 1) and soil characteristics. In each site, a pure *Q. ilex* stand (TP – Tirone Pure stand; SP – Somma Pure stand) and a mixed *Q. ilex*-*P. pinea* stand (TM – Tirone Mixed stand; SM – Somma Mixed stand) with comparable age of trees, soil and stand characteristics were sampled (Figure 1). The stands are forests and *P. pinea* trees were planted.

**Table 1:** Coordinates, altitude and structure features of the four selected stands.

	TP	TM	SP	SM
Latitude, longitude (°)	40.49050 N, 14.24124 E	40.812909 N, 14.402956 E	40.49902 N, 14.27067 E	40.832987 N, 14.454074 E
Altitude (m a.s.l.)	528	505	669	569
Mean *Q. ilex* stem diameter ± SE (cm)	34 ± 0.81	38 ± 1.49	24 ± 1.64	19 ± 1.67
Mean *Q. ilex* tree height ± SE (m)	16 ± 0.49	17 ± 0.48	13 ± 0.74	13 ± 0.82
*Q. ilex* stand density (tree/ha)	11000	10000	33000	9000
Total stand density (tree/ha)	11000	13000	33000	19000
Canopy cover (Leaf area index – LAI ± Standard error of the LAI determinations – SEL)	1.2 ± 0.07	1.69 ± 0.05	2.6 ± 0.04	2.25 ± 0.13
Slope (%)	20	0	50	100
Mean *P. pinea* stem diameter ± SE (cm)	–	53 ± 1.72	–	44 ± 1.74
Mean *P. pinea* tree height ± SE (m)	–	17 ± 0.45	–	16 ± 0.53


*TP and TM are the pure and the mixed stand, respectively, at the “Tirone Alto-Vesuvio” site, while SP and SM are the pure and the mixed stand, respectively, of the Mount Somma site.*

Both pure stands (TP and SP) are dominated by *Q. ilex* with an understory consisting in *Q. ilex* trees and the sporadic presence of *Robinia pseudoacacia* L., a non-native invasive species. Both mixed stands (TM and SM) are covered by *P. pinea* dominant trees with a *Q. ilex* understory and the sporadic presence of *R. pseudoacacia.* SP and SM stands are characterized by smaller trees and have higher stand density and steeper slope than TP and TM (Table 1). Moreover, *P. pinea* trees of the SM stand are taller than *Q. ilex* trees, while *P. pinea* and *Q. ilex* trees have a similar height at TM (Table 1). At the S site, total stand density and canopy cover are higher at the pure than at the mixed stand, while the slope is less steep at the pure than at the mixed one. At the T site, total stand density and canopy cover are lower while slope is steeper at the pure than at the mixed stand.

As regards soil, there were no significant differences in water content (WC), available water capacity (AWC) and water holding capacity (WHC) between the mixed and pure stand at each site. However, there were significant differences between the two sites in terms of AWC and WHC, with both the parameters higher at the T site (AWC mean value = 33.07 ± 12.87%; WHC mean value = 24.27 ± 7.33%) in comparison to S site (AWC mean value = 20.27 ± 5.33%; WHC mean value = 16.71 ± 3.79%) (*P* < 0.05). WC, AWC, and WHC were determined in autumn by taking six samples per site (three samples per each stand) and following standard procedures (USDA, 1996).

The climate is Mediterranean with dry summer and mild winter. Climate data of minimum, maximum and mean monthly temperature and total monthly precipitation from the nearest meteorological stations were interpolated and compared to the CRU TS3.23 gridded dataset at 0.5° resolution data (Harris et al., 2014). Since the correlation between the two data series was significant (as shown in Zalloni et al., 2018a), we used the CRU climate data for the analyses. Mean annual temperature and precipitation of the period 1985–2005 selected for statistical analysis are 16.4°C and 710 mm, respectively (Figure 1). The wettest month is November, with an average of 114 mm of cumulative precipitation, while the driest month is August, with an average of 24 mm of cumulative precipitation and the highest temperature of 30°C. The lowest mean temperatures are recorded in January, with an average of 9 °C (Figure 1). A dry season lasts from the middle of May to the end of August.

### Tree-Ring Growth Analysis

Two cores per tree were extracted at breast height from 20 dominant *Q. ilex* trees per stand in the “Tirone Alto-Vesuvio” site (T) and from 14 dominant *Q. ilex* trees per stand in the Mount Somma site (S). Being the sites in a Natural Park, the minimum number of trees to get a good EPS value was sampled. The cores were collected during September 2015 at the mixed sites, while during September 2016 at the pure ones. The number of cored trees per site is different because of the different availability of dominant trees. The cores were air dried, mounted on wooden supports and sanded. A Leica MS5 light microscope (Leica Microsystems, Germany) fitted with a LINTAB measuring system (Frank Rinn, Heidelberg, Germany) was used to measure ring-width chronologies with a resolution of 0.01 mm. After being visually cross-dated, tree-ring width chronologies were statistically checked with the TSAP-Win (Time Series Analysis and Presentation; Rinntech) and COFECHA (Holmes, 1983) softwares. Mean tree-ring width chronologies were developed per each stand. The Dendrochronology Program Library within the software R (dplR; Bunn, 2008, 2010) was used to calculate the expressed population signal (EPS) (Wigley et al., 1984), the mean RBAR (that is the is the mean correlation coefficient among tree-ring series) and the signal-to-noise ratio (SNR) in order to assess chronology quality (Table 2).

**Table 2:** Dendrochronological characteristics of *Q. ilex* tree-ring width chronologies of the four stands.

	TP	TM	SP	SM
Timespan	1948–2015	1949–2014	1966–2015	1965–2014
Lenght (years)	68	66	50	50
Tree-ring width (mm) (Mean value ± SE)	2.38 ± 0.16	2.62 ± 0.14	2.47 ± 0.12	2.36 ± 0.2
EPS^∗^	0.99	0.98	0.90	0.92
RBAR^∗∗^	0.86	0.70	0.45	0.50
SNR^∗∗∗^	118.84	42.84	9.62	11.13


*TP and TM are the pure and the mixed stand, respectively, at the “Tirone Alto-Vesuvio” site, while SP and SM are the pure and the mixed stand, respectively, at the Mount Somma site.^∗^Expressed population signal (EPS) is a measure of the common variability in a chronology and it is commonly acceptable for value >0.85; ^∗∗^RBAR is the mean correlation coefficient among tree-ring series, ranging from -1 to +1 (the higher the value, the stronger is the underlying common signal); ^∗∗∗^The signal-to-noise ratio (SNR) informs about the ratio between the signal (short-term variation) and the noises (long-term variation) contained in chronologies.*

In order to compare radial growth of the dominant trees between stands, correctly dated tree-ring width chronologies were converted into tree basal area increment (BAI) chronologies with the following formula:

(1) $$BAI_{t}=\pi R_{\left(t^{2}\right)}-\pi R_{\left(t-1^{2}\right)},$$

where *R*_t_ and *R*_t-1_ are the stem radius at the end and at the beginning of the annual increment, respectively, and *BAI*_t_ is the annual ring area at year t. Cumulative mean basal area was then calculated for each stand summing the average basal area. BAI instead of ring-width time series were chosen because they reduce tree-size and age effect on growth trends, keeping the high and low frequency signals of tree-ring width series at the same time (Tognetti et al., 2000; Biondi and Qeadan, 2008).

### Stable C and O Isotope Analysis

Five correctly dated cores of *Q. ilex* without defects per stand were chosen for isotopic analyses. Carbon and oxygen stable isotope analysis were conducted over the common period 1985–2005 for all the stands, where a change in wood growth was found between pure and mixed stands at both sites. Tree rings were manually split with annual resolution using a scalpel under a dissection microscope, and the derived samples of the five cores per species and per stand were then pooled together in order to maximize sample size. Preliminary analyses showed that comparable results are obtained by using either whole wood or cellulose (Borella et al., 1998; Korol et al., 1999; Barbour et al., 2001; Warren et al., 2001; Loader et al., 2003; Verheyden et al., 2005; Weigt et al., 2015), thus we decided to proceed on whole wood, without any chemical pre-treatment. The collected samples were milled with a centrifugal mill, weighted in silver capsules (aliquots of 0.8/1.0 mg) and pyrolyzed at 1450°C, (PYRO-cube, Elementar, Hanau, Germany). The annual δ^13^C and δ^18^O values of the obtained CO were determined simultaneously by a Delta Plus XP isotope ratio mass spectrometer (ThermoFinnigan MAT, Bremen, Germany) via a pyrolysis unit by a ConFlo III interface (ThermoFinnigan MAT). A subset of samples that covered the whole range of the expected δ^13^C values was measured again via oxygen combustion with an EA1110 elemental analyzer (CE Instruments, Milan, Italy) coupled to a Delta-S isotope ratio mass spectrometer (ThermoFinnigan MAT), in order to make a correction of the δ^13^C values. The δ^13^C signal obtained by pyrolysis is dampened because of “memory effects” compared to the more usually measured one obtained by oxygen combustion (Woodley et al., 2012). The formula used to correct the pyrolysis δ^13^C data was the following: δ^13^C_corr_ = 1.2526 × δ^13^C_pyro_ + 5.0032, where δ^13^C_corr_ is the corrected final δ^13^C value and δ^13^C_pyro_ is the value measured by pyrolysis and corrected with internal standards. Furthermore, δ^13^C values were corrected for the Suess effect, which is a shift in the atmospheric concentrations of carbon isotopes due to increasing fossil-fuel derived CO_2_ (Keeling, 1979). The corrected series were used for the subsequent statistical analyses.

### WUE_i_ Calculation From δ^13^C

Isotopic ^13^C-fractionation during CO_2_-fixation can be calculated as:

(2) $$\delta^{13}C_{\mathrm{plant}}=\delta^{13}C_{\mathrm{air}}-a-\left(b-a\right)*\left(c_{i}/c_{a}\right),$$

where *δ*^13^*C*_air_ is the carbon isotope ratio of atmospheric CO_2_, *a* is the fractionation factor due to CO_2_ diffusion through stomata (4.4%), *b* is the fractionation factor due to the Rubisco enzyme during photosynthesis (27.0‰), *c*_i_ is the intercellular leaf CO_2_ concentration, *c*_a_ is the atmospheric CO_2_ concentration and *δ*^13^*C*_plant_ is the carbon isotope ratio of plant organic matter, e.g., in tree-rings. WUE_i_ chronologies for each stand were then calculated following the formula reported by Ehleringer and Cerling (1995):

(3) $$WUE_{i}=A/g_{s}=\left(c_{a}-c_{i}\right)/1.6,$$

where *A* is the photosynthetic rate, *g*_s_ is the stomatal conductance and 1.6 is the ratio of diffusivity of water and CO_2_ in the atmosphere. This can be solved as *c*_i_ is known from Eq. (2). In particular, the following formula was used:

(2) $$WUE_{i}=\left(c_{a}-c_{i}\right)/1.6=\left[c_{a}-c_{a}\left(\Delta -a/b-a\right)\right]1/1.6=c_{a}\left[\left(1-\left(\Delta -a/b-a\right)\right)1/1.6\right],$$

where Δ is the carbon isotope discrimination which represents the difference between *δ*^13^*C*_air_ and *δ*^13^*C*_plant_, and using Eq. (1) *c*_i_ is equivalent to

(4) $$c_{a}\left[\left(\Delta -a\right)/\left(b-a\right)\right],$$

while *c*_a_ annual values were taken from the NOAA database (^1^Mauna Loa station). The parameter Δ was calculated as:

(5) $$\Delta =\left(\delta^{13}C_{\mathrm{air}}-\delta^{13}C_{\mathrm{plant}}\right)/\left(1+\delta^{13}C_{\mathrm{plant}}/1000\right).$$

*δ*^13^*C*_air_ values were taken from the ones estimated by McCarroll and Loader (2004) and the measured ones available online^2^, while *δ*^13^*C*_plant_ are the values measured in tree rings of our samples.

### Complementarity Calculations

In order to assess inter-specific facilitation and competition interactions for comparison of wood growth, WUE_i_ and δ^18^O of *Q. ilex* in pure and mixed stands, an annual index of complementarity was calculated for the period 1985-2005 for each site with the following formula (Forrester, 2015; Battipaglia et al., 2017):

(6) $$Complementarity\left(\%\right)=\left[\left(X_{M}-X_{P}\right)/X_{P})\right]*100,$$

where *X* is annual basal area, WUE_i_ or δ^18^O, *M* is related to mixed stands and *P* is related to pure stands. The index is positive when wood growth, WUE_i_ or δ^18^O are higher in mixed than in pure stands, while negative when they are higher in pure than in mixed stands.

To compare the two sites, in terms of WUE_i_, δ^18^O, and BAI, characterized by different number of samples, *U*-test was used through SPSS 13.0 statistical package (SPSS Inc., Chicago, IL, United States) (Spiegel, 1975).

### IADF Frequency Analysis

**FIGURE 2:**
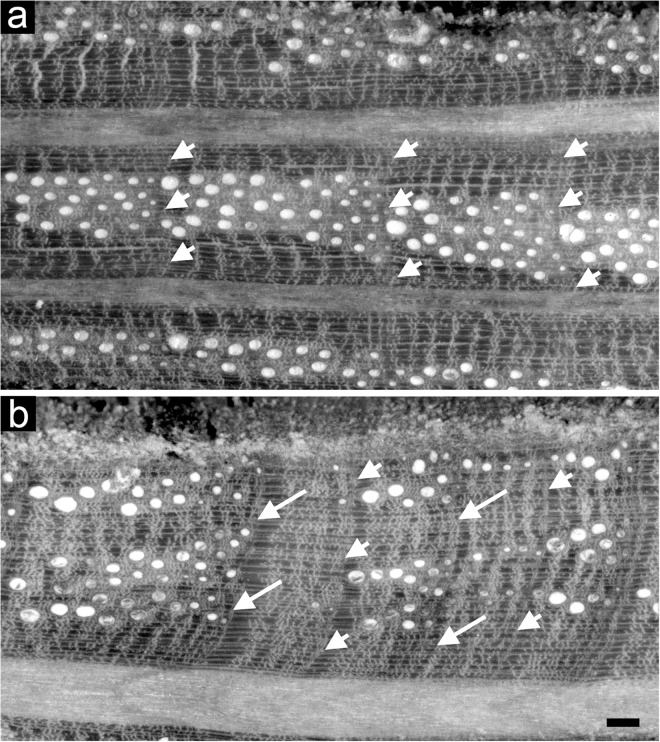
Light microscopy views of cross sections of tree rings of *Quercus ilex* without **(a)** and with IADFs **(b)**. Arrowheads point the boundaries of tree rings; arrows point to the IADF. Bar: 100 μm.

Intra-annual density fluctuation occurrence was detected within the rings of all the *Q. ilex* dated cores under a reflected light microscope. IADFs were identified by detecting variations in cell lumen area, frequency and wall density different from the “standard” transition from earlywood to latewood of *Q. ilex* described in Wheeler (2011), as found in Campelo et al. (2007) and defined in Zalloni et al. (2018b) (Figure 2). Relative annual IADF frequency chronologies of each stand were calculated as the ratio between the number of cores with an IADF and the total number of cores for each year. Stabilized annual IADF frequency chronologies were then calculated according to Osborn et al. (1997) as *f = F* ∗ *n*^0.5^ where *F* is the relative IADF frequency value and *n* is the total number of cores for each year, in order to stabilize the variance overcoming the problem of the changing sample depth over years. A percentage of IADF occurrence was calculated for each stand as the number of rings with IADF on the number of total rings for the period 1985–2005.

### Climate Analysis

The period 1985–2005 was selected for statistical analysis in order to match BAI data with isotope ones for comparisons, considering that in those years (more specifically 1996–1997) a change in wood growth was found between pure and mixed stands at both sites. In order to investigate the relations between growth traits and climate parameters, a Pearson’s linear correlation function analysis (*P* < 0.05) was implemented between cumulative mean annual BAI, WUE_i_, and δ^18^O annual values of the whole study period (1985–2005) and temperature and precipitation data. A Pearson’s linear correlation function analysis (*P* < 0.05) was also implemented between mean annual BAI of the period 1985–1996, mean annual BAI of the period 1997–2005 and temperature and precipitation data, in order to investigate whether and what climate factor significantly influenced tree growth at the pure and mixed stands of the two sites, and their ecophysiological responses. Temperature and precipitation data were seasonally grouped from December of the previous year to February of the next year, in order to certainly cover all the season (winter, spring, summer, autumn, and winter again) of the current year which could influence tree-ring growth in Mediterranean species (Cherubini et al., 2003; Vieira et al., 2015; Balzano et al., 2018). The analyses were performed using Excel^©^.

## Results

### Tree-Ring Growth, WUE_i_ and δ^18^O Trends

**FIGURE 3:**
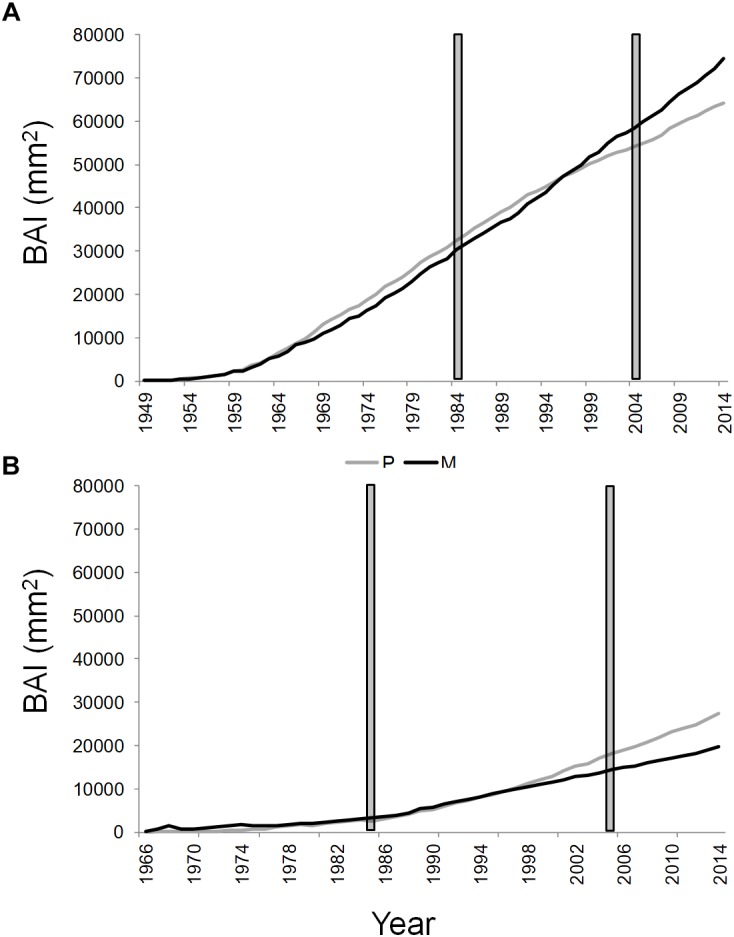
Cumulative basal area increment of *Q. ilex* growing in pure (in gray) and mixed (in black) stands, in the “Tirone Alto-Vesuvio” **(A)** and in the Mount Somma **(B)** sites. The gray bars delimit the period chosen for isotope and statistical analysis.

The dendrochronological characteristics of *Q. ilex* tree-ring width chronologies for the four stands are summarized in Table 2. Tree-ring chronologies of *Q. ilex* trees covered the timespan from 1949 to 2014 at the two stands of the T site, while the shorter timespan (from 1966 to 2014) was found at the S site (Table 2 and Figure 3).

The mean annual BAI for the period 1985–2005 of SM stand was the lowest (1063.31 ± 311.26 mm^2^, mean value ± standard error), followed by TP (1257.38 ± 360.67 mm^2^), SP (1274.51 ± 298.34 mm^2^), and TM stands (1665.08 ± 407.62 mm^2^).

The cumulative BAI of wood growth of the dominant trees of the stands revealed an opposite shift in wood growth of pure and mixed stands between the two sites from the year 1997 to 2014 (Figure 3). More specifically, a wood growth increase of dominant trees in the mixed compared to the pure stand was recorded at the T site (Figure 3A), while the opposite was found at the S site (Figure 3B). At the T and S sites, the basal area of *Q. ilex* accounted, respectively, for the 38.51 and 14.54% of the total basal area of the mixed stand.

**FIGURE 4:**
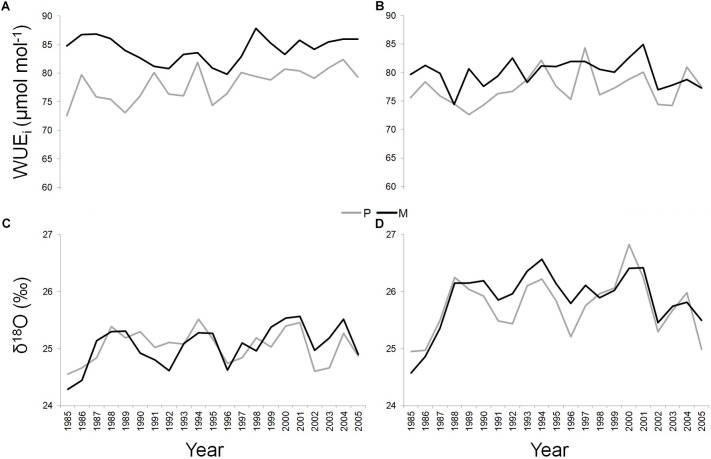
WUE_i_ and δ^18^O of *Q. ilex* growing in pure (in gray) and mixed (in black) stands for the period 1985–2005, at the “Tirone Alto-Vesuvio” **(A,C)** and at the Mount Somma **(B,D)** sites.

At the T site, WUE_i_ was significantly higher along the whole study period in the dominant trees of the mixed (mean value = 84.15 ± 2.22 μmol mol^-1^) compared to the pure stand (mean value = 78.04 ± 2.86 μmol mol^-1^) (*P* < 0.05) (Figure 4A). At the S site, this applied in the 71.43% of the cases (SM mean value = 79.91 ± 2.37 μmol mol^-1^; SP mean value = 77.2 ± 2.9 μmol mol^-1^) (*P* < 0.05) (Figure 4B).

At each site, δ^18^O values were similar between dominant trees growing in pure and mixed stands, while significantly absolute higher values of δ^18^O were found in both pure and mixed stand at the S site (SP mean value = 25.75 ± 0.49‰; SM mean value = 25.87 ± 0.5‰) compared to the T site (TP mean value = 25.04 ± 0.29‰; TM mean value = 25.05 ± 0.36‰) (*P* < 0.05) (Figure 4C,D).

Finally, a Pearson’s linear correlation function analysis (*P* < 0.05) implemented between WUE_i_ and δ^18^O values, showed a significant positive correlation only at the SM stand, during the period 1997–2005 (Pearson’s correlation value = 0.95).

### Complementarity Effects

**FIGURE 5:**
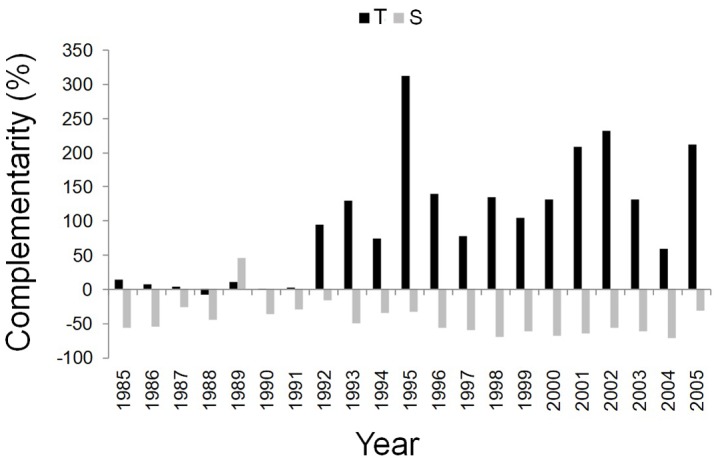
Temporal dynamics of complementarity effect for the annual basal area increment of *Q. ilex* growing in the pure stand compared to *Q. ilex* growing in the mixed stand for the period 1985–2005, at the “Tirone Alto-Vesuvio” (in black) and at the Mount Somma (in gray) sites.

After the year 1992, the complementarity effect analysis pointed out the difference between facilitation/competition interactions of the dominant trees of mixed and pure stands at the two sites. More specifically, from 1992 to 2005, *Q. ilex* wood growth was higher in the mixed than in the pure stand at the T site, while it was higher in the pure than in the mixed stand at the S site (Figure 5).

**FIGURE 6:**
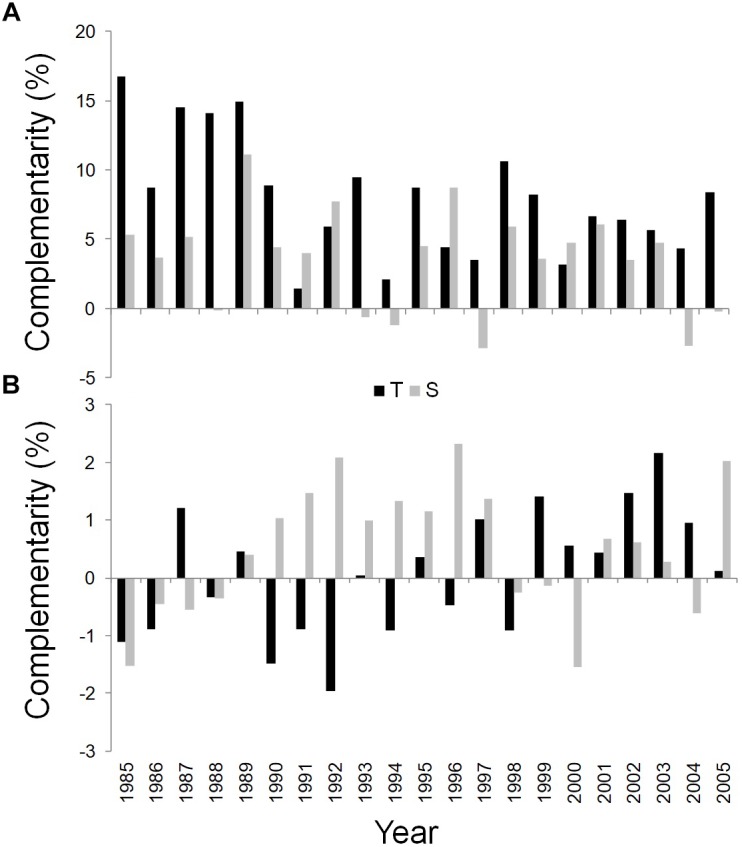
Temporal dynamics of complementarity effect for WUE_i_ **(A)** and δ^18^O **(B)** of *Q. ilex* growing in the pure stand compared to *Q. ilex* growing in the mixed stand for the period 1985–2005, at the Tirone Alto-Vesuvio (in black) and at the Mount Somma (in gray) sites.

WUE_i_ was found to be substantially higher in the dominant trees in mixed than in pure stands during the whole study period at both sites (Figure 6A).

Finally, the wood of the mixed stand is more enriched in δ^18^O than the pure one at the S site in most years, while an unclear pattern was shown for the complementarity index based on δ^18^O values of the T site (Figure 6B).

### IADF Frequency

The highest percentage of occurrence of IADFs in tree-rings of dominant *Q. ilex* trees, for the period 1985–2005, was found in both the stands of the S site. In particular, the highest one was recorded in the mixed stand of the S site (26.41%), followed by the pure one (16.61%), then the mixed stand of the T site (14.76%) followed by the pure one, which showed a very low IADF frequency (1.79%).

### Climate Influence

Climate analysis with cumulative mean annual BAI of the whole study period did not show significant correlations for any of the stands. The same lack of significant correlation was found for the analysis cumputed for the period 1985–1996. On the contrary, a significant influence of summer (from June to August) and autumn (from September to November) precipitation on cumulative mean annual BAI of the period 1997–2005 was found for all the stands, with higher Pearson’s coefficient values (r) for autumn (TP = 0.82; TM = 0.82; SP = 0.85; SM = 0.85) than for summer precipitation (TP = 0.69; TM = 0.71; SP = 0.71; SM = 0.71) (*P* < 0.05). Climate analysis with WUE_i_ and δ^18^O of the whole study period showed that precipitation was the main influencing factor. WUE_i_ was positively correlated with precipitation of winter of the previous year (from December of the previous year to February of the current year) and negatively with precipitation of current spring (from March to May) for the TM stand (*r* = 0.43 and -0.47, respectively), while negatively correlated with autumn precipitation for the SP stand (*r* = -0.45) (*P* < 0.05). δ^18^O was negatively driven by autumn precipitation for all the stands (*r* = -0.48 for TM; *r* = -0.57 for SP; *r* = -0.44 for SM) except for the TP one, where winter precipitation (from December of the current year to February of the next year) was the driving factor (*r* = -0.51) (*P* < 0.05). WUE_i_ was also positively correlated with summer temperature for the TM stand (*r* = 0.61) (*P* < 0.05).

## Discussion

### Different *Q. ilex* Wood Growth of Pure and Mixed Stands Within Each Site

Trends in *Q. ilex* cumulative BAI for the period 1985–2005, suggested different wood growth dynamics of trees growing at the two sites. More specifically, starting from the year 1997, the slope of wood growth trends were inverted between mixed and pure stands within each site; the mean BAI of *Q. ilex* of the mixed stand at the T site presented higher wood growth compared to the pure stand, while at the S site, the situation was exactly the opposite, with the higher growth of the pure stand than the mixed one. Such an inversion also fits well with the temporal variability observed in the BAI-based complementarity indexes. Summer and autumn precipitation seem to have driven the observed shift in wood growth between dominant trees in pure and mixed stands, since climate correlations with cumulative BAI showed no significant influences until 1996, while summer and autumn precipitation affected tree growth of all the stands starting from 1997. After the year 1996 a decrease in both summer and autumn precipitation accompanied by an increase in temperature is recorded (Supplementary Figure S1), which leads to drier conditions that have possibly triggered the complementarity interactions.

In water-limited Mediterranean ecosystems, water availability is the main factor affecting wood growth of *Q. ilex*, leading to changes in complementarity interactions, as shown by the high value of the BAI-based complementarity index at the T site associated with high summer precipitation in 1995. Therefore, with the occurrence of drier climatic conditions, at the T site an interaction effect of competitive reduction has been likely experienced, thus resulting in increased *Q. ilex* wood growth in the mixed than pure stand. This interaction, i.e., facilitation, between different species growing in the same stand supports several studies which found mixed stands with increased wood growth compared to monocoltures, being facilitated by segregation niche (including many processes like the inter-specific differences in phenology and physiology that reduce the competition for resources) (Roupsard et al., 1999; Moore et al., 2011; Schwendenmann et al., 2015). Different species growing in mixed stands also likely use different water sources due to differences in root architecture (Cherubini et al., 2003; Schume et al., 2004; Schwendenmann et al., 2015). In our study case, observed phenomena might be linked to different root systems with *Q. ilex* extracting water from deeper soil layers than *P. pinea*, or by different water use strategies. Indeed, the anisohydric species *Q. ilex* resists drought, thus behaving differently from the isohydric *P. pinea* which avoids drought to save water (Mayoral et al., 2015; Zalloni et al., 2018b). Differences in *Q. ilex* wood growth in pure and mixed stands, together with the occurrence of drier climatic conditions after 1997, were also found at the S site, even if with an opposite trend: competition outweighted any complementary effects in the mixed stand, with a reduced wood growth in *Q. ilex* compared to pure stand. Tougher conditions of growth with higher density and slope, and a soil with a lower WHC could have concurred to make *Q. ilex* more affected by *P. pinea* competitiveness at the S site. Moreover, stand density is in favor of *P. pinea* in SM stand. This assumption would be in contrast with the SGH, as well as with the CSR strategy theory, which suggest that facilitation in spite of competition increases between species when site conditions are harsher (Bertness and Callaway, 1994; Grime, 2007). However, it would instead agree with the resource-ratio theory described by Tilman (1985, 2007), which implies that inter-specific competition may be stronger where soil fertility and moisture is lower, as also showed by Trinder et al. (2012) for grassland species and by Coates et al. (2013) for *Picea glauca* (Moench) Voss associated with *Pinus contorta* Douglas ex Loudon, 1838. To further support this theory: Hunt et al. (1999) found that facilitation effects decreased with increasing stand density in *Eucalyptus nitens* H.Deane & Maiden stand mixed with *Acacia dealbata* Link, 1822 in Australia; del Río and Sterba (2009) showed a lower growth in mixed than in pure stands of *Pinus sylvestris* L., 1753 and *Quercus pyrenaica* Willd. in Spain driven by forest density. As a late successional species, *Q. ilex* at the pure stand at the S site could have increased growth compared to the pine-oak ecosystem (Crow, 1988; Urbieta et al., 2011), moving toward a state of climax community which is better adapted to stressed Mediterranean conditions of growth (Sheffer, 2012).

### Ecophysiological Responses of Pure and Mixed Stands of *Q. ilex*

Precipitation seems to be the most important limiting factor in controlling *Q.ilex* WUE_i_. Temperature showed only one significant correlation with WUE_i_, indicating little influence on inter-annual variations in water use efficiency. This is in agreement with several previous studies on *Quercus* species in the Mediterranean region (Ferrio et al., 2003; Ferrio and Voltas, 2005; Andreu et al., 2008; Maseyk et al., 2011). Autumn and winter precipitation seem to play a key role and represent the typical period for soil recharge in the Mediterranean area (Pumo et al., 2008).

The analysis of the WUE_i_ and the δ^18^O together with their relative complementarity indexes, revealed that *Q. ilex* dominant trees in mixed stands had a higher WUE_i_ at similar δ^18^O at both sites; moreover tree rings of dominant trees of both the stands at the S site were more enriched in ^18^O than those at the T one. This could indicate a tighter stomatal control in trees growing at the S than the T site, probably linked to its drier conditions with a soil characterized by less WHC thus a higher vapor pressure deficit at the leaf level (Roden and Ehleringer, 2000; Barbour et al., 2002). However, the higher δ^18^O at the S than at the T site could be also due to the fact that trees growing at the S site are younger and may rely on water (mainly precipitation) from upper soil layers, compared to the trees at the T site, which tend to capture less enriched water from deep soil horizons (Dawson et al., 2002). Further, a difference in WUE_i_ not associated with a difference in δ^18^O indicates that the high WUE_i_ observed in *Q. ilex* trees of the mixed stands was due to higher photosynthetic rates rather than lower stomatal conductance (Scheidegger et al., 2000). The processes that improve light and nutrient availability or uptake, which are driven by inter-specific differences in mixed stands, can enhance WUE_i_ enabling the plants to increase photosynthesis and make more efficient use of water resources (Forrester, 2015). Kunert et al. (2012) and Schwendenmann et al. (2015) found, respectively, a higher WUE in wood growth, calculated as the ratio between annual wood increment and water use, and a higher diversity in the water uptake depth in mixed stands than in monocoltures of tropical plants due to complementary water use. Forrester et al. (2010) showed an enhanced WUE due to increased N and P availability and light absorption in mixed stands which increased photosynthesis in *Eucalyptus globulus* growing with *Acacia mearnsii*. A high WUE_i_ could be associated with the high wood growth (Binkley et al., 2004; Binkley, 2012), as we found at the mixed stand at the T site. On the other hand, the higher WUEi found in the dominant trees in the mixed compared to the pure stand at the S site, did not determine an increase in tree growth, in agreement with other studies showing the lack of correlation between WUE_i_ and growth (Maseyk et al., 2011; Peñuelas et al., 2011; Battipaglia et al., 2013; Moreno-Gutiérrez et al., 2015), or even warming-induced growth reductions in spite of increasing WUE_i_ (Peñuelas et al., 2008; Linares and Camarero, 2012; Granda et al., 2014) for several Mediterranean species during drought periods. Indeed, carbon resources may be allocated to reproduction, to primary growth or just to other tissues such as roots (Dewar et al., 1994). During drought periods carbon investments in below-ground growth are in fact of higher priority than the above ground structures (Kotzlowski and Palladry, 2002) because below-ground growth is favored to guarantee water uptake (Saxe et al., 1998). The decrease in *Q. ilex* wood growth at the SM stand, although the enrichment of WUE_i_, could be due to reduced stomatal conductance after increasing warming-related drought, as Brito et al. (2016) showed for *P. canariensis* in Spain. Indeed, the positive correlation found between δ^13^C-derived WUE_i_ and δ^18^O for dominant trees growing at SM, suggests that *g*_s_ played a significant role (Scheidegger et al., 2000; Moreno-Gutiérrez et al., 2012). According to the observed cumulative BAI reduction, the less favorable growth conditions at the S site, with a higher tree density, a steeper slope and a lower soil WHC of the topsoil, could have concurred to intensify the drought-induced stomatal closure reducing transpiration in the mixed stand, at the price of reducing net assimilation rate, as Brito et al. (2014) showed for *P. canariensis* at a treeline site with low soil WHC. *Q. ilex* trees growing in the mixed stand at the S site were probably more affected by competition, given that *P. pinea* trees presence prevailed. Furthermore, young Mediterranean trees could be more sensitive to limiting climatic conditions than older ones (Rozas et al., 2009, 2013; Vieira et al., 2009; Brito et al., 2016; Zalloni et al., 2016), confirming the hypothesis that the younger *Q. ilex* trees at the S site suffered from competition with *P. pinea* rather than being benefited from facilitation. Coherently, WUE_i_ and δ^18^O-based complementarity indexes showed that competition prevailed over facilitation for dominant trees in the mixed stand at the S site, where the higher WUE_i_ was, however, accompained by higher ^18^O ratios compared to trees in the pure stand, suggesting a tighter stomatal control of *Q. ilex* mixed with *P. pinea*, which was not shown for *Q. ilex* growing alone. Conte et al. (2018) found that *Fagus sylvatica* growing in a mixed stand with *P. sylvestris* had high WUEi but low productivity not only due to competition but also due to other factors, such as nutrient limitation and forest management.

The highest percentage of IADFs was found in tree rings of *Q. ilex* dominant plants growing at the S site, where harsher growth conditions, due to the higher stand density, steeper slope, a soil with a lower WHC, and a tighter stomatal control, were observed. A higher frequency of IADFs in tree rings enriched in ^18^O at a site with drier growth conditions, compared to a wetter site, was also found in *Erica arborea* L. tree-rings by Battipaglia et al. (2014), showing that the formation of these peculiar wood anatomical traits is an indicator of the ability of trees to face stressful conditions. Furthermore, within the two sites, more IADFs occurred in tree rings in dominant trees in the mixed stands compared to the ones in the pure ones: the high IADF occurrence could thus be also related to the higher WUE_i_ recorded in tree rings of *Q. ilex* growing at the mixed stands. A high WUE_i_ is often influencing the ability of a species to withstand water stress (Battipaglia et al., 2014), and interpreted as an adaptation to drought-prone environments (Raven, 2002). In this view, the higher IADF frequency in tree rings of mixed stands than pure ones, accompanying the higher WUE_i_, is a further support that IADFs should be considered a sign of the ability of a species to avoid stress conditions; in such a way, a positive carbon balance under dry conditions would be maintained through a high WUE, regardless of differences in wood growth.

## Conclusion

The observed differences between wood growth of *Q. ilex* dominant trees in pure and mixed stands growing at two sites, highlighted the importance of local site conditions in determining the inter- and intra-specific interactions underlying the growth response to environmental variability. The occurrence of drier climatic conditions from 1997 was shown to trigger opposite complementarity interactions for *Q. ilex* growing with *P. pinea* trees at the two sites characterized by different soil WHC, stand density and slope. Competitive reduction was experienced at the site with higher soil WHC, lower stand density and less steep slope, while competition became a limiting factor at the other site. WUE_i_ increased in trees of both mixed stands at the two sites, but the isotopes showed completely different ecophysiological processes behind tree growth. At the T site, the increase in WUE_i_ was mainly related to higher photosynthetic rates that lead to an increase in wood growth. Differently, at the S site, WUE_i_ increase was related to a more conservative strategy saving water through stomata closure, thus not leading to wood growth increase. IADF frequency in *Q. ilex* tree-rings seemed to be linked to stressful conditions rather than to favorable ones, and could be interpreted as an adaptation aimed at avoiding dry periods, independently from wood growth differences. The analysis of a combination of different tree-ring parameters helped to find plausible physiological causes of the observed interactions. The findings of this study case highlight the importance of considering site conditions in planning forest management strategies in the view of forecasted increase in water shortage for mixed and pure forests of *Q. ilex* and *P. pinea*. Based on our results, at those specific sites, thinnings of *P. pinea* mixed stands with *Q. ilex*, where trees are young and stand density is high, could be a good choice to limit inter-specific competition for resources and to promote *Q. ilex* wood growth. On the contrary, when good conditions of stand density are present, promoting the co-existence of *Q. ilex* and *P. pinea* could facilitate complementarity in resource use, while thinning pure *Q. ilex* stands could limit intra-specific competition. To draw general strategies in planning forest management, further case studies, which also take dominated trees into account, are needed. These would help assessing the influence of stand structure, soil and environmental conditions on complementarity interactions in Mediterranean *Q. ilex* mixed stands, also analyzing IADF occurrence as an indicator of species capability to avoid stressful conditions.

## Data Availability

The raw data supporting the conclusions of this manuscript will be made available by the authors, without undue reservation, to any qualified researcher.

## Author Contributions

EZ, GB, and VDM conceived and designed the study. EZ performed sampling and analyses and wrote the main part of the manuscript. GB, VDM, PC, and MS contributed to the analyses. All authors contributed to interpretation of the overall data and wrote specific parts, made a critical revision of the whole text, and approved the submitted version of the manuscript.

## Conflict of Interest Statement

The authors declare that the research was conducted in the absence of any commercial or financial relationships that could be construed as a potential conflict of interest.

## Footnotes

1 http://www.esrl.noaa.gov/

2 http://www.esrl.noaa.gov/gmd/

## Supplementary Material

FIGURE S1 Summer (from June to August) **(a)** and autumn (from September to November) **(b)** total precipitation (in blue) and mean temperature (in red) of the CRU TS3.23 gridded dataset at 0.5° resolution data (Harris et al., 2014) for the period 1985–2005. The gray bar indicates a decrease in both summer and autumn precipitation accompanied by an increase in temperature after the year 1996.

## References

[B1] Altieri S. Mereu S. Cherubini P. Castaldi S. Sirignano C. Lubritto C. (2015). Tree-ring carbon and oxygen isotopes indicate different water use strategies in three Mediterranean shrubs at Capo Caccia (Sardinia, Italy). *Trees* 29 1593–1603. 10.1007/s00468-015-1242-z

[B2] Amoroso M. M. Turnblom E. C. (2006). Comparing productivity of pure and mixed Douglas-fir and western hemlock plantations in the Pacific Northwest. *Can. J. For. Res.* 36 1484–1496. 10.1139/x06-042

[B3] Andreu L. Planells O. Gutierrez E. Helle G. Schleser G. H. (2008). Climatic significance of tree-ring width and δ13C in a Spanish pine forest network. *Tellus B Chem. Phys. Meteorol.* 60 771–781. 10.1111/j.1600-0889.2008.00370.x

[B4] Balzano A. Èufar K. Battipaglia G. Merela M. Prislan P. Aronne G. (2018). Xylogenesis reveals the genesis and ecological signal of IADFs in *Pinus pinea* L. and *Arbutus unedo* L. *Ann. Bot.* 121 1231–1242. 10.1093/aob/mcy008 29415209 PMC5946860

[B5] Barbour M. M. Andrews T. J. Farquhar G. D. (2001). Correlations between oxygen isotope ratios of wood constituents of *Quercus* and *Pinus* samples from around the world. *Aust. J. Plant Physiol.* 28 335–348. 10.1071/PP00083

[B6] Barbour M. M. Walcroft A. S. Farquhar G. D. (2002). Seasonal variation in delta C-13 and delta O-18 of cellulose from growth rings of *Pinus radiata*. *Plant Cell Environ.* 25 1483–1499. 10.1046/j.0016-8025.2002.00931.x

[B7] Battipaglia G. Campelo F. Vieira J. Grabner M. De Micco V. Nabais C. (2016a). Structure and function of intra–annual density fluctuations: mind the gaps. *Front. Plant Sci.* 7:595. 10.3389/fpls.2016.00595 27200063 PMC4858752

[B8] Battipaglia G. De Micco V. Brand W. A. Saurer M. Aronne G. Linke P. (2014). Drought impact on water use efficiency and intra-annual density fluctuations in *Erica arborea* on Elba (Italy). *Plant Cell Environ.* 37 382–391. 10.1111/pce.12160 23848555

[B9] Battipaglia G. Savi T. Ascoli D. Castagneri D. Esposito A. Mayr S. (2016b). Effects of prescribed burning on ecophysiological, anatomical and stem hydraulic properties in *Pinus pinea* L. *Tree Physiol.* 36 1019–1031. 10.1093/treephys/tpw034 27178842

[B10] Battipaglia G. Pelleri F. Lombardi F. Altieri S. Vitone A. Conte E. (2017). Effects of associating *Quercus robur* L. and *Alnus cordata* Loisel. on plantation productivity and water use efficiency. *For. Ecol. Manage.* 391 106–114. 10.1016/j.foreco.2017.02.019

[B11] Battipaglia G. Saurer M. Cherubini P. Calfapietra C. McCarthy H. R. Norby R. J. (2013). Elevated CO2 increases tree-level intrinsic water use efficiency: insights from carbon and oxygen isotope analyses in tree rings across three forest FACE sites. *New Phytol.* 197 544–554. 10.1111/nph.12044 23215904

[B12] Bertness M. D. Callaway R. M. (1994). Positive interactions in communities. *Trends Ecol. Evol.* 9 191–193. 10.1016/0169-5347(94)90088-4 21236818

[B13] Binkley D. (2003). Seven decades of stand development in mixed and pure stands of conifers and nitrogen-fixing red alder. *Can. J. For. Res.* 33 2274–2279. 10.1139/x03-158

[B14] Binkley D. (2012). “Understanding the role resource use efficiency in determining the growth of trees and forests,” in *Forests in Development: A Vital Balance*, eds Schlichter T. Montes L. (Berlin: Springer), 13–26.

[B15] Binkley D. Stape J. L. Ryan M. G. (2004). Thinking about efficiency of resource use in forests. *For. Ecol. Manage.* 193 5–16. 10.1016/j.foreco.2004.01.019

[B16] Biondi F. Qeadan F. (2008). A theory-driven approach to tree-ring standardization: defining the biological trend from expected basal area increment. *Tree-Ring Res.* 64 81–96. 10.3959/2008-6.1

[B17] Borella S. Leuenberger M. Saurer M. Siegwolf R. (1998). Reducing uncertainties in δ13C analysis of tree rings: pooling, milling, and cellulose extraction. *J. Geophys. Res.* 103 19519–19526. 10.1029/98JD01169

[B18] Brienen R. J. W. Gloor E. Clerici S. Newton R. Arppe L. Boom A. (2017). Tree height strongly affects estimates of water-use efficiency responses to climate and CO_2_ using isotopes. *Nat. Commun.* 8:288. 10.1038/s41467-017-00225-z 28819277 PMC5561090

[B19] Brito P. Grams T. E. E. Matysssek R. Jimenez M. S. Gonzalez-Rodríguez A. M. Oberhuber W. (2016). Increased water use efficiency does not prevent growth decline of *Pinus canariensis* in a semi-arid treeline ecotone in Tenerife, Canary Islands (Spain). *Ann. For. Sci.* 73 741–749. 10.1007/s13595-016-0562-5 27482149 PMC4961253

[B20] Brito P. Lorenzo J. R. Gonzalez-Rodríguez A. M. Morales D. Wieser G. Jiménez M. S. (2014). Canopy transpiration of a *Pinus canariensis* forest at the tree line: implications for its distribution under predicted climate warming. *Eur. J. For. Res.* 133 491–500. 10.1007/s10342-014-0779-5

[B21] Brooker R. W. (2006). Plant-plant interactions and environmental change. *New Phytol.* 171 271–284. 10.1111/j.1469-8137.2006.01752.x 16866935

[B22] Bunn A. G. (2008). A dendrochronology program library in R (dplR). *Dendrochronologia* 26 115–124. 10.1016/j.dendro.2008.01.002

[B23] Bunn A. G. (2010). Statistical and visual crossdating in R using the dplR library. *Dendrochronologia* 28 251–258. 10.1016/j.dendro.2009.12.001

[B24] Campelo F. Gutiérrez E. Ribas M. Nabais C. Freitas H. (2007). Relationships between climate and double rings in Quercus ilex from northeast Spain. *Can. J. For. Res.* 37 1915–1923. 10.1139/X07-050

[B25] Cherubini P. Gartner B. L. Tognetti R. Bräker O. U. Schoch W. Innes J. L. (2003). Identification, measurement and interpretation of tree rings in woody species from Mediterranean climates. *Biol. Rev.* 78 119–148. 10.1017/S1464793102006000 12620063

[B26] Coates K. D. Lilles E. B. Astrup R. (2013). Competitive interactions across a soil fertility gradient in a multispecies forest. *J. Ecol.* 101 806–818. 10.1111/1365-2745.12072

[B27] Conte E. Lombardi F. Battipaglia G. Palombo C. Altieri S. La Porta N. (2018). Growth dynamics, climate sensitivity and water use efficiency in pure vs. mixed pine and beech stands in Trentino (Italy). *For. Ecol. Manage.* 409 707–718. 10.1016/j.foreco.2017.12.011

[B28] Crow T. R. (1988). Reproductive mode and mechanisms for selfreplacement of northern red oak (Quercus rubra)-a review. *For. Sci.* 34 19–40.

[B29] Čufar K. (2007). Dendrochronology and past human activity – A review of avances since 2000. *Tree-Ring Res.* 63 47–60. 10.3959/1536-1098-63.1.47 25217744

[B30] Dawson T. E. Mambelli S. Plamboeck A. H. Templer P. H. Tu K. P. (2002). Stable isotopes in plant ecology. *Annu. Rev. Ecol. Evol. Syst.* 33 507–559. 10.1146/annurev.ecolsys.33.020602.095451

[B31] De Micco V. Campelo F. de Luis M. Bräuning A. Grabner M. Battipaglia G. (2016). Formation of intra-annual-density-fluctuations in tree rings: how, when, where and why? *IAWA J.* 37 232–259. 10.1163/22941932-20160132

[B32] del Río M. Schütze G. Pretzsch H. (2014). Temporal variation of competition and facilitation in mixed species forests in Central Europe. *Plant Biol.* 16 166–176. 10.1111/plb.12029 23581485

[B33] del Río M. Sterba H. (2009). Comparing volume growth in pure and mixed stands of *Pinus sylvestris* and *Quercus pyrenaica*. *Ann. For. Sci.* 66:502 10.1051/forest/2009035

[B34] Dewar R. C. Ludlow A. R. Dougherty P. M. (1994). Environmental influences on carbon allocation in pines. *Ecol. Bull.* 43 92–101.

[B35] Dieler J. Pretzsch H. (2013). Morphological plasticity of European beech (*Fagus sylvatica* L.) in pure and mixed-species stands. *For. Ecol. Manage.* 295 97–108. 10.1016/j.foreco.2012.12.049

[B36] Ehleringer J. R. Cerling T. E. (1995). Atmospheric CO_2_ and the ratio of intercellular to ambient CO_2_ concentrations in plants. *Tree Physiol.* 15 105–111. 10.1093/treephys/15.2.105 14965982

[B37] Erickson H. E. Harrington C. A. Marshall D. D. (2009). Tree growth at stand and individual scales in two dual-species mixture experiments in southern Washington State, USA. *Can. J. For. Res.* 39 1119–1132. 10.1139/X09-040

[B38] Ferrio J. P. Florit A. Vega A. Serrano L. Voltas J. (2003). δ13C and tree-ring width reflect different drought responses in Quercus ilex and Pinus halepensis. *Oecologia* 137 512–518. 10.1007/s00442-003-1372-7 14505023

[B39] Ferrio J. P. Voltas J. (2005). Carbon and oxygen isotope ratios in wood constituents of *Pinus halepensis* as indicators of precipitation, temperature and vapour pressure deficit. *Tellus B Chem. Phys. Meteorol.* 57 164–173. 10.1111/j.1600-0889.2005.00137.x

[B40] Fonti P. von Arx G. García-González I. Eilmann B. Sass-Klaassen U. Gärtner H. (2010). Studying global change through investigation of the plastic responses of xylem anatomy in tree rings. *New Phytol.* 185 42–53. 10.1111/j.1469-8137.2009.03030.x 19780986

[B41] Forrester D. I. (2014). The spatial and temporal dynamics of species interactions in mixed-species forests: from pattern to process. *For. Ecol. Manage.* 312 282–292. 10.1016/j.foreco.2013.10.003

[B42] Forrester D. I. (2015). Transpiration and water-use efficiency in mixed-species forests versus monocultures: effects of tree size, stand density and season. *Tree Physiol.* 35 289–304. 10.1093/treephys/tpv011 25732385

[B43] Forrester D. I. Kohnle U. Albrecht A. T. Bauhus J. (2013). Complementarity in mixed-species stands of *Abies alba* and *Picea abies* varies with climate, site quality and stand density. *For. Ecol. Manage.* 304 233–242. 10.1016/j.foreco.2013.04.038

[B44] Forrester D. I. Theiveyanathan S. Collopy J. J. Marcar N. E. (2010). Enhanced water use efficiency in a mixed *Eucalyptus globulus* and *Acacia mearnsii* plantation. *For. Ecol. Manage.* 259 1761–1770. 10.1016/j.foreco.2009.07.036

[B45] Gessler A. Ferrio J. P. Hommel R. Treydte K. Werner R. A. Monson R. K. (2014). Stable isotopes in tree rings: towards a mechanistic understanding of isotope fractionation and mixing processes from the leaves to the wood. *Tree Physiol.* 34 796–818. 10.1093/treephys/tpu040 24907466

[B46] Giorgi F. (2006). Climate change hot-spots. *Geophys. Res. Lett.* 33 L08707. 10.1029/2006GL025734

[B47] Granda E. Rosattop D. R. Camarero J. J. Voltas J. Valladares F. (2014). Growth and carbon isotopes of Mediterranean trees reveal contrasting responses to increased carbon dioxide and drought. *Oecoloia* 174 307–317. 10.1007/s00442-013-2742-4 23928889

[B48] Grime J. P. (2007). Plant strategy theories: a comment on Craine (2005). *J. Ecol.* 95 227–230. 10.1111/j.1365-2745.2006.01163.x

[B49] Grossiord C. Granier A. Gessler A. Jucker T. Bonal D. (2014a). Does drought influence the relationship between biodiversity and ecosystem functioning in boreal forests? *Ecosystems* 17 394–404. 10.1007/s10021-013-9729-1

[B50] Grossiord C. Granier A. Ratcliffe S. Bouriaud O. Bruelheide H. Cheæko E. (2014b). Tree diversity does not always improve resistance of forest ecosystems to drought. *Proc. Natl. Acad. Sci. U.S.A.* 111 14812–14815. 10.1073/pnas.1411970111 25267642 PMC4205672

[B51] Grossiord C. Granier A. Gessler A. Pollastrini M. Bussotti F. Bonal D. (2014c). Interspecific competition influences the response of oak transpiration to increasing drought stress in a mixed Mediterranean forest. *For. Ecol. Manage.* 318 54–61. 10.1016/j.foreco.2014.01.004

[B52] Harris I. Jones P. D. Osborn T. J. Lister D. H. (2014). Updated high-resolution grids of monthly climatic observations – The CRU TS3.10 Dataset. *Int. J. Climatol.* 34 623–642. 10.1002/joc.3711

[B53] Holmes R. L. (1983). Computer-assisted quality control in tree ring dating and measurement. *Tree Ring Bull.* 43 69–78. 30197912

[B54] Hunt M. A. Unwin G. L. Beadle C. L. (1999). Effects of naturally regenerated *Acacia dealbata* on the productivity of a *Eucalyptus nitens* plantation in Tasmania, Australia. *For. Ecol. Manage.* 117 75–85. 10.1016/S0378-1127(98)00467-8

[B55] Huxman T. E. Barron-Gafford G. Gerst K. L. Angert A. L. Tyler A. P. Venable D. L. (2008). Photosynthetic resource-use efficiency and demographic variability in desert winter annual plants. *Ecology* 89 1554–1563. 10.1890/06-2080.1 18589520

[B56] IPCC (2017). “Meeting report of the intergovernmental panel on climate change expert meeting on mitigation, sustainability and climate stabilization scenarios,” in *IPCC Working Group III Technical Support Unit*, eds Shukla P.R.J. Skea R. van Diemen K. Calvin Ø. Christophersen F. Creutzig J. (London: Imperial College London).

[B57] Kahle D. Wickham H. (2013). ggmap: spatial visualization with ggplot2. *R J.* 5 144–161. 10.32614/RJ-2013-014

[B58] Keeling C. D. (1979). The suess effect: 13Carbon-14Carbon interrelations. *Environ. Int.* 2 229–300. 10.1016/0160-4120(79)90005-9

[B59] Korol R. L. Kirschbaum M. U. F. Farquhar G. D. Jeffreys M. (1999). Effects of water status and soil fertility on the C-isotope signature in *Pinus radiata*. *Tree Physiol.* 19 551–562. 10.1093/treephys/19.9.551 12651529

[B60] Kotzlowski T. Palladry S. (2002). Acclimation and adaptive responses of woody plants to environmental stress. *Bot. Rev.* 68 270–334. 10.1663/0006-8101(2002)068[0270:AAAROW]2.0.CO;2 12102516

[B61] Kunert N. Schwendenmann L. Potvin C. Hölscher D. (2012). Tree diversity enhances tree transpiration in a Panamanian forest plantation. *J. Appl. Ecol.* 49 135–144. 10.1111/j.1365-2664.2011.02065.x

[B62] Law B. E. Falge E. Gu L. Baldocchi D. D. Bakwin P. Berbigier P. (2002). Environmental controls over carbon dioxide and water vapor exchange of terrestrial vegetation. *Agric. For. Meteorol.* 113 97–120. 10.1016/S0168-1923(02)00104-1

[B63] Lebourgeois F. Gomez N. Pinto P. Mérian P. (2013). Mixed stands reduce *Abies alba* tree-ring sensitivity to summer drought in the Vosges mountains, western Europe. *For. Ecol. Manage.* 303 61–71. 10.1016/j.foreco.2013.04.003

[B64] Linares J. C. Camarero J. J. (2012). From pattern to process: linking intrinsic water-use efficiency to drought-induced forest decline. *Glob. Chang. Biol.* 18 1000–1015. 10.1111/j.1365-2486.2011.02566.x

[B65] Loader N. J. Robertson I. McCarroll D. (2003). Comparison of stable carbon isotope ratios in the whole wood, cellulose and lignin of oak tree-rings. *Palaeogeogr. Palaeoclimatol. Palaeoecol.* 196 395–407. 10.1016/S0031-0182(03)00466-8

[B66] Maseyk K. Hemming D. Angert A. Leavitt S. W. Yakir D. (2011). Increase in water-use efficiency and underlying processes in pine forests across a precipitation gradient in the dry Mediterranean region over the past 30 years. *Oecologia* 167 573–585. 10.1007/s00442-011-2010-4 21590331

[B67] Mayoral C. Calama R. Sánchez-González M. Pardos M. (2015). Modelling the influence of light, water and temperature on photosynthesis in young trees of mixed Mediterranean forests. *New For.* 46 485–506. 10.1007/s11056-015-9471-y

[B68] McCarroll D. Loader N. J. (2004). Stable isotopes in tree rings. *Quat. Sci. Rev.* 23 771–801. 10.1016/j.quascirev.2003.06.017

[B69] Moore G. W. Bond B. J. Jones J. A. (2011). A comparison of annual transpiration and productivity in monoculture and mixed-species douglas-fir and red alder stands. *For. Ecol. Manage.* 262 2263–2270. 10.1016/j.foreco.2011.08.018

[B70] Moreno-Gutiérrez C. Battipaglia G. Cherubini P. Delgado Huertas A. Querejeta J. I. (2015). Pine afforestation decreases the long-term performance of understorey shrubs in a semi-arid Mediterranean ecosystem: a stable isotope approach. *Funct. Ecol.* 29 15–25. 10.1111/1365-2435.12311

[B71] Moreno-Gutiérrez C. Dawson T. E. Nicolás E. Querejeta J. I. (2012). Isotopes reveal contrasting water use strategies among coexisting plant species in a Mediterranean ecosystem. *New Phytol.* 196 489–496. 10.1111/j.1469-8137.2012.04276.x 22913668

[B72] Osborn T. J. Briffa K. R. Jones P. D. (1997). Adjusting variance for sample-size in tree-ring chronologies and other regional-mean time-series. *Dendrochronologia* 15 89–99.

[B73] Peñuelas J. Canadell J. G. Ogaya R. (2011). Increased water-use efficiency during the 20th century did not translate into enhanced tree growth. *Glob. Ecol. Biogeogr.* 20 597–608. 10.1111/j.1466-8238.2010.00608.x

[B74] Peñuelas J. Hunt J. M. Ogaya R. Jump A. S. (2008). Twentieth century changes of tree-ring δ13C at the southern range-edge of *Fagus sylvatica*: increasing water-use efficiency does not avoid the growth decline induced by warming at low altitudes. *Glob. Chang. Biol.* 14 1076–1088. 10.1111/j.1365-2486.2008.01563.x

[B75] Pretzsch H. Block J. Dieler J. Dong P. H. Kohnle U. Nagel J. (2010). Comparison between the productivity of pure and mixed stands of Norway spruce and European beech along an ecological gradient. *Ann. For. Sci.* 76 712–723. 10.1051/forest/2010037

[B76] Pretzsch H. Bielak K. Block J. Bruchwald A. Dieler J. Ehrhart H. P. (2013a). Productivity of mixed versus pure stands of oak (*Quercus petraea* (MATT.) LIEBL. and *Quercus robur* L.) and European beech (*Fagus sylvatica* L.) along an ecological gradient. *Eur. J. For. Res.* 132 263–280. 10.1007/s10342-012-0673-y

[B77] Pretzsch H. Schütze G. Uhl E. (2013b). Resistance of European tree species to drought stress in mixed versus pure forests: evidence of stress release by interspecific facilitation. *Plant Biol.* 15 483–495. 10.1111/j.1438-8677.2012.00670.x 23062025

[B78] Pumo D. Viola F. Noto L. V. (2008). Ecohydrology in Mediterranean areas: a numerical model to describe growing seasons out of phase with precipitations. *Hydrol. Earth Syst. Sci.* 12 303–316. 10.5194/hess-12-303-2008

[B79] Raven J. A. (2002). Selection pressures on stomatal evolution. *New Phytol.* 153 371–386. 10.1046/j.0028-646X.2001.00334.x 33863217

[B80] Roden J. S. Ehleringer J. R. (2000). Hydrogen and oxygen isotope ratios of tree ring cellulose for field-grown riparian trees. *Oecoloia* 123 481–489. 10.1007/s004420000349 28308756

[B81] Roupsard O. Ferhi A. Granier A. Pallo F. Depommier D. Mallet B. (1999). Reverse phenology and dry-season water uptake by *Faidherbia albida* (Del.) A. Chev. in an agroforestry parkland of Sudanese west Africa. *Funct. Ecol.* 13 460–472. 10.1046/j.1365-2435.1999.00345.x

[B82] Rozas V. DeSoto L. Olano J. M. (2009). Sex-specific, age depedent sensitivity of tree-ring growth to climate in the deciduous tree Juniperus thurifera. *New Phytol.* 182 687–697. 10.1111/j.1469-8137.2009.02770.x 19210720

[B83] Rozas V. Garcia-Gonzales I. Perez-de-Lis G. (2013). Local and large-scale climatic factors controlling tree-ring growth of Pinus canariensis on an oceanic island. *Clim. Res.* 56 197–207. 10.3354/cr01158

[B84] Saxe H. Ellsworth D. S. Heath J. (1998). Trees and forest functioning in an enriched CO_2_ atmosphere. *New Phytol.* 139 395–436. 10.1046/j.1469-8137.1998.00221.x

[B85] Scheidegger Y. Saurer M. Bahn M. Siegwolf R. T. W. (2000). Linking stable oxygen and carbon isotopes with stomatal conductance and photosynthetic capacity: a conceptual model. *Oecologia* 125 350–357. 10.1007/s004420000466 28547329

[B86] Schume H. Jost G. Hager H. (2004). Soil water depletion and recharge patterns in mixed and pure forest stands of European beech and Norway spruce. *J. Hydrol.* 289 258–274. 10.1016/j.jhydrol.2003.11.036

[B87] Schwendenmann L. Pendall E. Sanchez-Bragado R. Kunert N. Hölscher D. (2015). Tree water uptake in a tropical plantation varying in tree diversity: interspecific differences, seasonal shifts and complementarity. *Ecohydrology* 8 1–12. 10.1002/eco.1479

[B88] Sheffer E. (2012). A review of the development of Mediterranean pine-oak ecosystems after land abandonment and afforestation: are they novel ecosystems? *Ann. For. Sci.* 69 429–443. 10.1007/s13595-011-0181-0

[B89] Somot S. Sevault F. Déqué M. Crépon M. (2008). 21th century climate change scenario for the Mediterranean using a coupled atmosphere-ocean regional climate model. *Glob. Planet. Change* 63 112–126. 10.1016/j.gloplacha.2007.10.003

[B90] Spiegel M. R. (1975). *Schaum’s Outline of Theory and Problems of Probability and Statistics.* New York, NY: McGraw-Hil.

[B91] Terradas J. (1999). “Holm oak and holm oak forests: an introduction,” in *Forest Ecology and Management*, eds Attiwill P. Binkley D. Fredericksen T. S. Laclau J.-P. Sterba H. (Berlin: Springer), 3–14.

[B92] Tilman D. (1985). The resource-ratio hypothesis of plant succession. *Am. Nat.* 125 827–852. 10.1086/284382

[B93] Tilman D. (2007). Resource competition and plant traits: a response to Craine et al. 2005. *J. Ecol.* 95 231–234. 10.1111/j.1365-2745.2007.01201.x

[B94] Tognetti R. Cherubini P. Innes J. L. (2000). Comparative stem-growth rates of Mediterranean trees under background and naturally-enhanced ambient CO_2_ concentrations. *New Phytol.* 146 59–74. 10.1046/j.1469-8137.2000.00620.x

[B95] Trinder C. J. Brooker R. W. Davidson H. Robinson D. (2012). A new hammer to crack and old nut: interspecific competitive resource capture by plants is regulated by nutrient supply, not climate. *PLoS One* 7:e29413. 10.1371/journal.pone.0029413 22247775 PMC3256146

[B96] Urbieta I. R. García L. V. Zavala M. A. Marañón T. (2011). Mediterranean pine and oak distribution in southern Spain: is there a mismatch between regeneration and adult distribution? *J. Veg. Sci.* 22 18–31. 10.1111/j.1654-1103.2010.01222.x

[B97] USDA, Natural Resources Conservation Service, and National Soil Survey Center (1996). *Soil survey Laboratory Methods Manual.* Berkshire: Books Express Publishing.

[B98] Verheyden A. Roggeman M. Bouillon S. Elskens M. Beeckman H. Koedam N. (2005). Comparison between δ13C of a-cellulose and bulk wood in the mangrove tree Rhizophora mucronata: implications for dendrochemistry. *Chem. Geol.* 219 275–282. 10.1016/j.chemgeo.2005.02.015

[B99] Vieira J. Campelo F. Nabais C. (2009). Age-dependent responses of treering growth and intra-annual density fluctuations of *Pinus pinaster* to Mediterranean climate. *Trees* 23 257–265. 10.1007/s00468-008-0273-0

[B100] Vieira J. Campelo F. Rossi S. Carvalho A. Freitas H. Nabais C. (2015). Adjustment capacity of maritime pine cambial activity in drought-prone environments. *PLoS One* 10:126223. 10.1371/journal.pone.0126223 25961843 PMC4427410

[B101] Warren C. R. McGrath J. F. Adams M. A. (2001). Water availability and carbon isotope discrimination in conifers. *Oecologia* 127 476–486. 10.1007/s004420000609 28547484

[B102] Weigt R. B. Bräunlich S. Zimmermann L. Saurer M. Grams T. E. E. Dietrich H. (2015). Comparison of δ18O and δ13C values between tree-ring whole wood and cellulose in five species growing under two different site conditions. *Rapid Commun. Mass Spectrom.* 29 2233–2244. 10.1002/rcm.7388 26522315

[B103] Wheeler E. A. (2011). InsideWood - a web resource for hardwood anatomy. *IAWA J.* 32 199–211. 10.1163/22941932-90000051

[B104] Wigley T. M. L. Briffa K. R. Jones P. D. (1984). On the average value of correlated time series, with applications in dendroclimatology and hydrometeorology. *J. Clim. Appl. Meteorol.* 23 201–213. 10.1175/1520-0450(1984)023<0201:OTAVOC>2.0.CO;2

[B105] Woodley E. J. Loader N. J. McCarroll D. Young G. H. F. Robertson I. Heaton T. H. E. (2012). High-temperature pyrolysis/gas chromatography/isotope ratio mass spectrometry: simultaneous measurement of the stable isotopes of oxygen and carbon in cellulose. *Rapid Commun. Mass Spectrom.* 26 109–114. 10.1002/rcm.5302 22173798

[B106] Zalloni E. Battipaglia G. Cherubini P. De Micco V. (2018a). Site conditions influence the climate signal of intra-annual density fluctuations in tree rings of Q. ilex L. *Ann. For. Sci.* 75–68. 10.1007/s13595-018-0748-0

[B107] Zalloni E. Battipaglia G. Cherubini P. Saurer M. De Micco V. (2018b). Contrasting physiological responses to Mediterranean climate variability are revealed by intra-annual density fluctuations in tree rings of *Quercus ilex* L. and *Pinus pinea* L. *Tree Physiol.* 38 1–12. 10.1093/treephys/tpy061 29920596

[B108] Zalloni E. de Luis M. Campelo F. Novak K. De Micco V. di Filippo A. (2016). Climatic signals from intra-annual density fluctuation frequency in Mediterranean pines at a regional scale. *Front. Plant Sci.* 7:579. 10.3389/fpls.2016.00579 27200052 PMC4852653

