# Supplementary figures and images for: Wood Growth in Pure and Mixed Quercus ilex L. Forests: Drought Influence Depends on Site Conditions
Source: Front Plant Sci. 2019 Apr 2;10:397. doi: 10.3389/fpls.2019.00397 (PMC6454142; doi:10.3389/fpls.2019.00397)

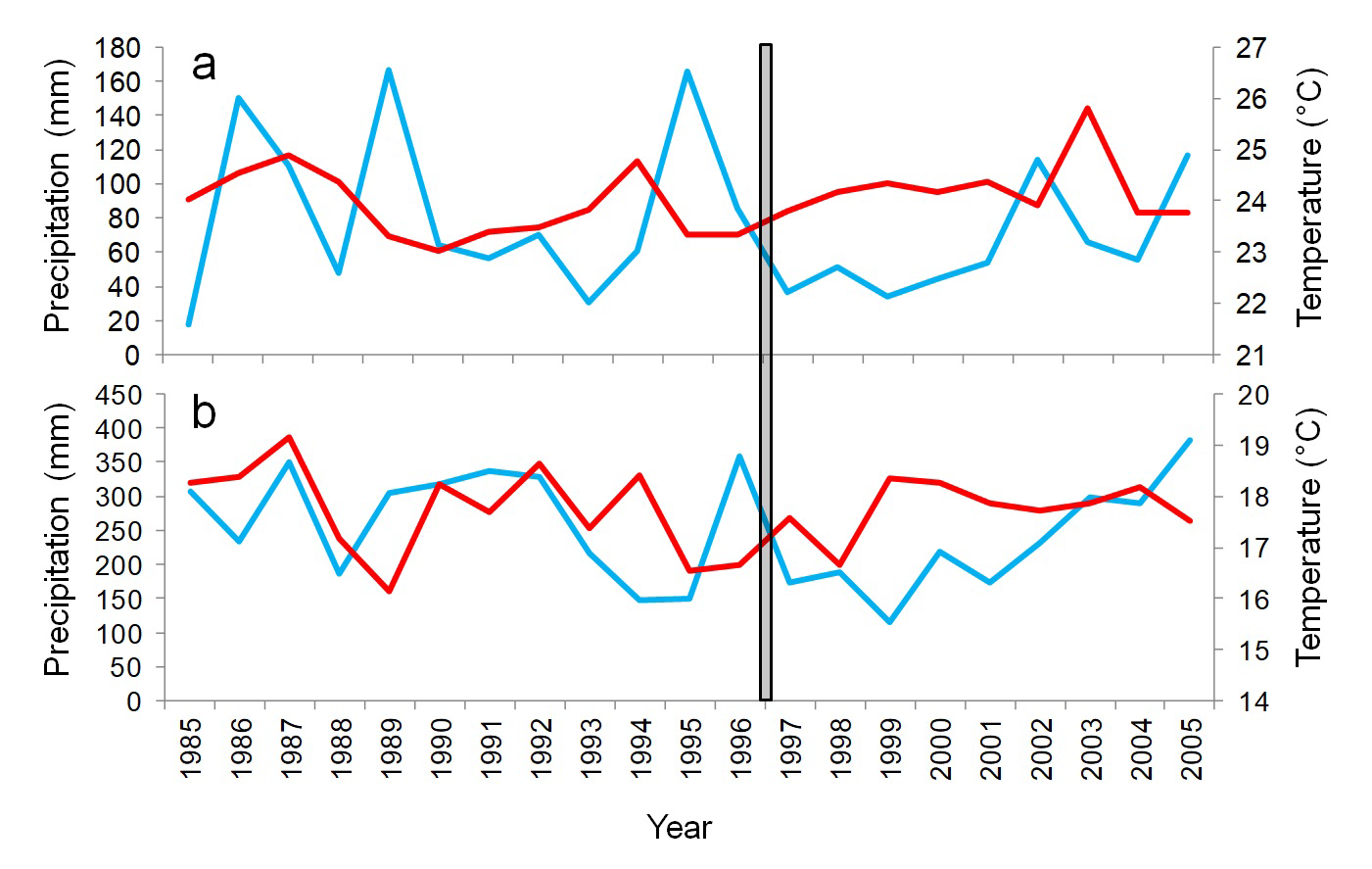

Supplement: FIGURE S1 — Summer (from June to August) (a) and autumn (from September to November) (b) total precipitation (in blue) and mean temperature (in red) of the CRU TS3.23 gridded dataset at 0.5° resolution data (Harris et al., 2014) for the period 1985–2005. The gray bar indicates a decrease in both summer and autumn precipitation accompanied by an increase in temperature after the year 1996. [file Image_1.TIF]
